# Socio‐economic inequalities in second primary cancer incidence: A competing risks analysis of women with breast cancer in England between 2000 and 2018

**DOI:** 10.1002/ijc.35320

**Published:** 2025-01-24

**Authors:** Ruchika Golani, Eva Kagenaar, Jérémie Jégu, Aurélien Belot, Suping Ling

**Affiliations:** ^1^ Inequalities in Cancer Outcomes Network (ICON) group, Department of Health Services Research and Policy, Faculty of Public Health and Policy London School of Hygiene & Tropical Medicine London UK; ^2^ Faculty of Naturopathy and Yogic Sciences SGT University Gurugram India; ^3^ Département d'Information Médicale Groupe Hospitalier Saint Vincent Strasbourg France

**Keywords:** breast cancer, competing risks, flexible parametric survival model, second primary cancer, socio‐economic inequalities

## Abstract

We aimed to investigate socio‐economic inequalities in second primary cancer (SPC) incidence among breast cancer survivors. Using Data from cancer registries in England, we included all women diagnosed with a first primary breast cancer (PBC) between 2000 and 2018 and aged between 18 and 99 years and followed them up from 6 months after the PBC diagnosis until a SPC event, death, or right censoring, whichever came first. We used flexible parametric survival models adjusting for age and year of PBC diagnosis, ethnicity, PBC tumour stage, comorbidity, and PBC treatments to model the cause‐specific hazards of SPC incidence and death according to income deprivation, and then estimated standardised cumulative incidences of SPC by deprivation, taking death as the competing event. Multiple imputation was performed to account for missing data. Among 649,905 included women, 47,399 SPCs and 171,223 deaths occurred during 4,269,042 person‐years of follow‐up. Income deprivation was consistently associated with an increased rate of SPC incidence (cause‐specific hazard ratio for the most vs. least deprived quintile: 1.29; 95% CI: 1.25, 1.33) and of death (1.36; 1.34, 1.38), translating into an absolute risk difference (the most vs. least deprived quintile) of 1.3% (95% CI: 1.0, 1.5) for SPC incidence and 4.9% (95% CI: 4.6, 5.1) for death at 10 years. Women with PBC from deprived areas in England faced a substantially higher risk of SPC than their counterparts. Future research is warranted to understand mechanisms for observed inequalities to inform strategies to monitor, prevent, and identify SPC in women with PBC.

## What's New?

Breast cancer incidence tends to be higher in women with higher socio‐economic status, but with a lower mortality rate. Whether socio‐economic inequalities also affect second primary cancers remains unclear. In this study using data from cancer registries and accounting for all‐cause death as the competing event, income deprivation was associated with increased rates of second primary cancer incidence and death. Of 55,000 new breast cancer cases every year, more than 600 cases of second primary cancer could be avoided if inequalities were eliminated. Strategies to monitor, prevent and identify second primary cancers should be in place to minimise disparities.

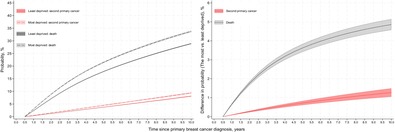

## 1 INTRODUCTION

Breast cancer is the most common cancer in women in the United Kingdom and worldwide, with approximately 55,500 newly diagnosed cases each year between 2016 and 2018,^1^ and the latest estimate of 2.3 million new cases in 2022 worldwide.^2^ Advancements in breast cancer screening, diagnostic techniques, and treatment regimens have resulted in improved survival—on average, 76% of women in England now survive for a decade or more.^1^ However, this journey of survivorship is not without its challenges, and the risk of developing second primary cancer (SPC) is a significant concern for cancer survivors, which could be attributed to the interplay of diverse factors ranging from genetic predisposition to environmental exposure, hormones, and treatment modalities.^3^, ^4^, ^5^

Several epidemiological studies from the United States,^6^, ^7^, ^8^ Europe, Canada, Australia, Singapore,^5^ Japan,^9^ Spain,^10^ and Israel,^11^ and meta‐analyses have reported a higher risk of SPC in women living with breast cancer compared to the general population,^12^, ^13^, ^14^ and such risk may vary by menopausal status—higher in women diagnosed with the primary breast cancer (PBC) before than after menopause,^12^ or by Continents—higher in Asians than in Europeans.^13^

Data on SPC after PBC diagnosis from England are scarce. In addition, inequalities in breast cancer incidence and mortality are well established—people with higher socio‐economic status tended to have higher breast cancer incidence but lower mortality rate^15^—but less is known for SPC incidence in women who already had a breast cancer diagnosis. While most studies on SPC focused on comparisons with the general population, it is unclear whether there are differences in SPC incidence by deprivation, given the longstanding socio‐economic inequalities in breast cancer survival.^16^ Therefore, we aimed to address this gap by examining socio‐economic inequalities in SPC incidence in England, while considering all‐cause death as the competing risk.

## 2 METHODS

### 2.1 Data source and population

We used the National Cancer Registration and Analysis Services (NCRAS) from NHS Digital to construct a cohort of individuals with breast cancer diagnosis. The NCRAS database is the population‐wide national registry for neoplasms in England and includes information on socio‐demographic factors and receipt of treatment.^17^ The NCRAS database is linked to the Office for National Statistics (ONS) death registration to obtain information on vital status, and Hospital Episodes Statistics Admitted Patient Care (HES APC) on procedures and diagnoses.

All women diagnosed with first ever Breast Cancer (ICD‐10: C50) between 1 January 2000 and 30 June 2018, aged between 18 and 99 years at diagnosis, and no prior cancer history were included in the analysis.

### 2.2 Exposure, outcomes, and covariates

We linked our cohort to Indices of Multiple Deprivation 2015 (IMD 2015) at lower super output area (LSOA) level using the patient's residence at the time of PBC diagnosis. We measured deprivation using the Income Domain from IMD 2015, which was based on the proportion of the population in an LSOA experiencing deprivation related to low income (benefit claims).^18^

The primary outcome was the diagnosis of SPC, and the competing outcome was death that occurred during the follow‐up (and before the occurrence of a SPC). SPC was defined as any subsequent malignant cancer diagnosis excluding non‐melanoma skin cancers (ICD‐10 codes: C00–C97 except C44) and with a different ICD‐10 code of their PBC. SPC types were categorised into cancer of second breast (C50, if the last digit was different to their PBC), digestive (C15–C26), female genital organ (C51–C58), respiratory and intrathoracic organ (C30–C39), and others. As cancer records within 6 months after their PBC diagnosis date were still likely to be related to their PBC, we followed up all women from 183 days after their PBC diagnosis date (this diagnosis date +183 days hereafter being referred to as index date) and excluded those who died or were lost to follow‐up before the index date.^19^ All women were followed up until the occurrence of SPC, death or right censoring date (31 December 2018), whichever occurred first.

Demographic factors included age, year of PBC diagnosis, and ethnicity. Year of PBC diagnosis was categorised as 2000–2003, 2004–2007, 2008–2011, 2012–2014, and 2015–2018. Ethnicity was from the NCRAS and classified into White, Asian, Black, Other Ethnic Group. Clinical factors included treatment types (curative surgery, radiotherapy, chemotherapy, and hormone therapy; all as binary—Yes/No—variables), the presence of a comorbidity (binary), and tumour stage (Stages 1–4, and missing if applicable). We extracted treatment information from NCRAS between 1 month before the PBC diagnosis and index date; we also supplemented the radiotherapy records from the National Radiotherapy Dataset. Comorbidity, defined as any disease listed in Charlson Comorbidity index, was identified from HES APC within 6 months and 6 years before the index date.^20^

### 2.3 Statistical analysis

Characteristics of included women by income deprivation were reported as median and interquartile range (IQR) for continuous variables, and number and percentage for categorical variables. We calculated crude incidence rates of overall SPC, specific SPC groups, and death in the study population overall, as well as by socio‐economic deprivation. We estimated the non‐parametric cumulative incidence of overall SPC considering death as a competing event, and of each specific SPC group considering other SPCs (in addition to death) as competing events.

For both overall SPC incidence and death, we applied a Royston–Parmar flexible parametric model with time from index date as time scale and a spline with five knots for the log‐cumulative hazard.^21^ The regression analyses for both death and overall SPC incidence were adjusted for demographic (age, year of PBC diagnosis, and ethnicity) and clinical factors (tumour stage, comorbidities, and treatment), in which age was transformed with a cubic spline with four knots (at 5th, 35th, 65th, and 95th) to allow non‐linear effects. Standardised cumulative incidences were estimated after cause‐specific regression modelling^22^ to contrast the risk of overall SPC by income deprivation, considering death as the competing event. To account for missing data in ethnicity and stage in our analysis, multiple imputation (10 times) was performed and both cause‐specific hazard ratios (CSHRs) and cumulative incidences were combined with Rubin's rules.^23^

We conducted several sensitivity analyses to assess the robustness of our multiple imputation results. First, since stage was the main missing variable, we treated missing stage as a missing category, and analysed people with complete data on all other variables (sensitivity analysis 1). Second, we conducted the complete‐case analysis (sensitivity analysis 2). Third, we considered second primary non‐breast cancer as the primary outcome. As NCRAS was improving their records of staging by calendar year, and the proportion of missing was reduced to nearly 10% by 2012, we further stratified our multiple imputation analysis by year of diagnosis before or after 2012, to assess whether the proportion of missing stage would affect the validity of our multiple imputation analyses. In addition, we also stratified our analysis by age of diagnosis, younger or older than 55 years, as a proxy of menopause, to examine whether the risk of SPC associated with income deprivation differed by pre‐ and post‐menopausal PBC. We also conducted sensitivity analyses by censoring all women at 1, 3, 5, 10, and 15 years after the index date. Lastly, as there were some debates around the definition of SPC for cancer of paired organs (such as breast cancer),^24^ we reported 10‐year cumulative risk of SPC derived from non‐parametric Aalen‐Johansen estimator using different definitions. Analysis was conducted with Stata MP 18.0 (StataCorp LLC, TX) and 95% confidence intervals (CIs) were reported for all estimates.

## 3 RESULTS

A total of 690,445 individuals were diagnosed with a PBC in our study period. Of them, 40,540 were excluded due to non‐female cases, invalid records of idiagnosis date, date of birth, or vital status, being diagnosed via death certificate, aged under 18 or over 100 years, or not under observation at index date, leaving the final cohort of 649,905 women for all the analyses (Figure S1).

### 3.1 Baseline characteristics

The characteristics of the included women by income quintile are presented in Table 1. The median age of PBC diagnosis was 62.0 years (IQR: 51.5–72.9). Most women were diagnosed with Stage II breast cancer (38.2%), reported their ethnicity as White (89.3%), and received curative surgery (73.9%). The most common combination of treatment was curative surgery with radiotherapy (29.0%), followed by curative surgery alone (20.9%) or curative surgery with chemotherapy (20.8%). The distribution of baseline characteristics was similar across income quintiles, but compared to the least deprived, slightly more women from the most deprived group were diagnosed at very young or very old age (e.g., the most vs. least deprived for women, same below, diagnosed between 18.0 and 44.9 years: 12.6% vs. 10.4%; between 75.0 and 99.9 years: 22.1% vs. 19.2%), reported as Asian or Black ethnicity (e.g., Asian 5.0% vs. 1.6%) and comorbidity (4.7% vs. 2.4%). In terms of treatment, the least deprived tended to receive more curative surgery (71.2% vs. 76.1%) and radiotherapy (33.5% vs. 38.5%) while the most deprived chemotherapy (32.2% vs. 30.4%).

**TABLE 1:** Subject characteristics on the date of diagnosis of primary breast cancer (PBC) by income quintiles.

	1—the least deprived	2	3	4	5—The most deprived	Total
	*N* = 149,720	*N* = 147,535	*N* = 135,107	*N* = 118,451	*N* = 99,092	N = 649,905
Year of diagnosis						
2000–2003	29,391 (19.6%)	28,735 (19.5%)	26,632 (19.7%)	23,505 (19.8%)	19,989 (20.2%)	128,252 (19.7%)
2004–2007	31,110 (20.8%)	30,723 (20.8%)	28,352 (21.0%)	24,979 (21.1%)	20,768 (21.0%)	135,932 (20.9%)
2008–2011	32,320 (21.6%)	32,186 (21.8%)	29,206 (21.6%)	25,588 (21.6%)	21,379 (21.6%)	140,679 (21.6%)
2012–2014	26,071 (17.4%)	25,611 (17.4%)	23,424 (17.3%)	20,151 (17.0%)	16,903 (17.1%)	112,160 (17.3%)
2015–2018	30,828 (20.6%)	30,280 (20.5%)	27,493 (20.3%)	24,228 (20.5%)	20,053 (20.2%)	132,882 (20.4%)
Age at PBC diagnosis, years						
Median (IQR)	61.5 (51.4–71.6)	62.3 (51.9–72.8)	62.5 (51.8–73.3)	62.2 (51.4–73.6)	61.7 (50.9–73.3)	62.0 (51.5–72.9)
18.0–44.9	15,642 (10.4%)	14,918 (10.1%)	14,539 (10.8%)	13,784 (11.6%)	12,499 (12.6%)	71,382 (11.0%)
45.0–54.9	34,752 (23.2%)	32,532 (22.1%)	29,059 (21.5%)	25,598 (21.6%)	21,886 (22.1%)	143,827 (22.1%)
55.0–64.9	38,367 (25.6%)	36,648 (24.8%)	32,454 (24.0%)	27,812 (23.5%)	23,079 (23.3%)	158,360 (24.4%)
65.0–74.9	32,157 (21.5%)	32,258 (21.9%)	28,952 (21.4%)	24,354 (20.6%)	19,695 (19.9%)	137,416 (21.1%)
75.0–99.9	28,802 (19.2%)	31,179 (21.1%)	30,103 (22.3%)	26,903 (22.7%)	21,933 (22.1%)	138,920 (21.4%)
Ethnicity						
White	135,767 (90.7%)	134,996 (91.5%)	121,599 (90.0%)	103,659 (87.5%)	84,477 (85.3%)	580,498 (89.3%)
Asian	2324 (1.6%)	2381 (1.6%)	3706 (2.7%)	4748 (4.0%)	4924 (5.0%)	18,083 (2.8%)
Black	503 (0.3%)	761 (0.5%)	1638 (1.2%)	3228 (2.7%)	4417 (4.5%)	10,547 (1.6%)
Other	1629 (1.1%)	1629 (1.1%)	1890 (1.4%)	2056 (1.7%)	2052 (2.1%)	9256 (1.4%)
Missing	9497 (6.3%)	7768 (5.3%)	6274 (4.6%)	4760 (4.0%)	3222 (3.3%)	31,521 (4.9%)
Stage						
I	28,105 (18.8%)	27,564 (18.7%)	24,386 (18.0%)	20,019 (16.9%)	17,545 (17.7%)	117,619 (18.1%)
II	56,833 (38.0%)	57,269 (38.8%)	51,628 (38.2%)	44,821 (37.8%)	37,579 (37.9%)	248,130 (38.2%)
III	8638 (5.8%)	9160 (6.2%)	8578 (6.3%)	8054 (6.8%)	7139 (7.2%)	41,569 (6.4%)
IV	3977 (2.7%)	4371 (3.0%)	4077 (3.0%)	3932 (3.3%)	3540 (3.6%)	19,897 (3.1%)
Missing	52,167 (34.8%)	49,171 (33.3%)	46,438 (34.4%)	41,625 (35.1%)	33,289 (33.6%)	222,690 (34.3%)
Comorbidity						
No	146,176 (97.6%)	143,254 (97.1%)	130,513 (96.6%)	113,942 (96.2%)	94,480 (95.3%)	628,365 (96.7%)
Yes	3544 (2.4%)	4281 (2.9%)	4594 (3.4%)	4509 (3.8%)	4612 (4.7%)	21,540 (3.3%)
Curative surgery						
No	35,745 (23.9%)	36,504 (24.7%)	35,596 (26.3%)	33,269 (28.1%)	28,568 (28.8%)	169,682 (26.1%)
Yes	113,975 (76.1%)	111,031 (75.3%)	99,511 (73.7%)	85,182 (71.9%)	70,524 (71.2%)	480,223 (73.9%)
Radiotherapy						
No	92,021 (61.5%)	92,297 (62.6%)	86,197 (63.8%)	77,105 (65.1%)	65,876 (66.5%)	413,496 (63.6%)
Yes	57,699 (38.5%)	55,238 (37.4%)	48,910 (36.2%)	41,346 (34.9%)	33,216 (33.5%)	236,409 (36.4%)
Chemotherapy						
No	104,199 (69.6%)	102,735 (69.6%)	93,793 (69.4%)	81,273 (68.6%)	67,225 (67.8%)	449,225 (69.1%)
Yes	45,521 (30.4%)	44,800 (30.4%)	41,314 (30.6%)	37,178 (31.4%)	31,867 (32.2%)	200,680 (30.9%)
Treatment overview						
Curative surgery only	32,430 (21.7%)	31,278 (21.2%)	28,150 (20.8%)	23,996 (20.3%)	20,025 (20.2%)	135,879 (20.9%)
Curative surgery with radiotherapy	45,593 (30.5%)	44,318 (30.0%)	39,155 (29.0%)	32,859 (27.7%)	26,262 (26.5%)	188,187 (29.0%)
Curative surgery with chemotherapy	30,516 (20.4%)	30,666 (20.8%)	28,022 (20.7%)	24,694 (20.8%)	21,067 (21.3%)	134,965 (20.8%)
Curative surgery with radiotherapy and chemotherapy	5436 (3.6%)	4769 (3.2%)	4184 (3.1%)	3633 (3.1%)	3170 (3.2%)	21,192 (3.3%)
Radiotherapy only	5063 (3.4%)	4572 (3.1%)	4178 (3.1%)	3547 (3.0%)	2719 (2.7%)	20,079 (3.1%)
Chemotherapy only	7962 (5.3%)	7786 (5.3%)	7715 (5.7%)	7544 (6.4%)	6565 (6.6%)	37,572 (5.8%)
Radiotherapy and chemotherapy	1607 (1.1%)	1579 (1.1%)	1393 (1.0%)	1307 (1.1%)	1065 (1.1%)	6951 (1.1%)
No cancer‐directed treatment	21,113 (14.1%)	22,567 (15.3%)	22,310 (16.5%)	20,871 (17.6%)	18,219 (18.4%)	105,080 (16.2%)
Hormone therapy						
No	97,594 (65.2%)	93,032 (63.1%)	84,826 (62.8%)	74,066 (62.5%)	62,817 (63.4%)	412,335 (63.4%)
Yes	52,126 (34.8%)	54,503 (36.9%)	50,281 (37.2%)	44,385 (37.5%)	36,275 (36.6%)	237,570 (36.6%)

Abbreviation: IQR, interquartile range.

Information on the stage of diagnosis and ethnicity was missing for 34.3% and 4.9% of the women, respectively. The baseline characteristics of women with complete and missing data are presented in Table S1.

### 3.2 Crude SPC incidence rates and all‐cause mortality rate

Table 2 shows the number of events, crude SPC incidence rates, and all‐cause mortality rate for all women and those from the least and most deprived areas. Among 4,269,0242 person‐years, 171,223 deaths and 47,399 SPCs occurred. The crude mortality rate and overall SPC incidence rate in our study population were 40.11 (39.92, 40.30) and 11.10 (95% CI: 11.00, 11.20) per 1000 person‐years, respectively, over the whole study period (Table 2). The most common SPC was cancer of the digestive organs (rate: 2.65; 95% CI: 2.60, 2.70), followed by second breast cancer (rate: 2.20; 95% CI: 2.16, 2.25). Compared to the least deprived quintile, women from the most deprived areas had a higher all‐cause mortality rate [50.36 (95% CI: 49.80, 50.92) vs. 32.51 (95% CI: 32.16, 32.86)] and SPC incidence rate [12.71 (95% CI: 12.43, 13.00) vs. 10.19 (95% CI: 9.99, 10.38)], in which the largest difference was observed for second cancer of respiratory and intrathoracic organs (2.84 [95% CI: 2.71, 2.98] vs. 1.13 [95% CI: 1.07, 1.20]), followed by digestive organs (2.93 [95% CI: 2.80, 3.07] vs. 2.50 [95% CI: 2.41, 2.60]). Minimal differences were observed for second breast and other cancer incidence rates (Table 2). Crude rates for other quintiles are reported in Table S2.

**TABLE 2:** The number of events and crude rates for SPC and death in the whole cohort and in the least and most deprived quintile of income.

	Overall (Person‐years: 4,269,042)		The least deprived (Person‐years: 1,028,432)		The most deprived (Person‐years: 613,557)	
	Events	Incidence rate	Events	Incidence rate	Events	Incidence rate
Overall second primary cancer	47,399	11.10 (11.00, 11.20)	10,477	10.19 (9.99, 10.38)	7799	12.71 (12.43, 13.00)
Second primary breast cancer (C50)	9403	2.20 (2.16, 2.25)	2214	2.15 (2.06, 2.24)	1368	2.23 (2.11, 2.35)
Second primary cancer female genital organs (C51–C58)	7382	1.73 (1.69, 1.77)	1633	1.59 (1.51, 1.67)	1139	1.86 (1.75, 1.97)
Second primary cancer of digestive organs (C15–C26)	11,306	2.65 (2.60, 2.70)	2572	2.50 (2.41, 2.60)	1800	2.93 (2.80, 3.07)
Second primary cancer of respiratory and intrathoracic organs (C30–C39)	7119	1.67 (1.63, 1.71)	1163	1.13 (1.07, 1.20)	1745	2.84 (2.71, 2.98)
Other second primary cancers	12,189	2.86 (2.80, 2.91)	2895	2.81 (2.71, 2.92)	1747	2.85 (2.72, 2.98)
Death	171,223	40.11 (39.92, 40.30)	33,433	32.51 (32.16, 32.86)	30,898	50.36 (49.80, 50.92)

*Note*: Incidence rate is per 1000 person‐years.

**FIGURE 1:**
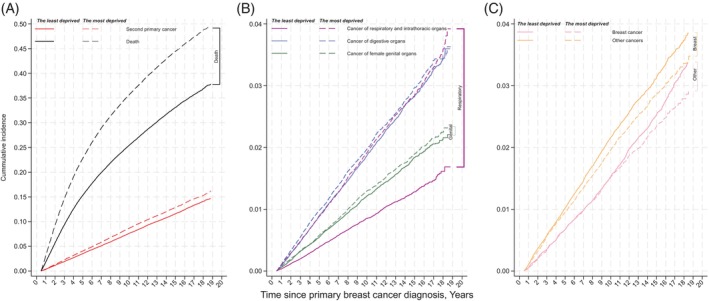
The non‐parametric cumulative incidence of second primary cancer (overall and by subgroups) and death in the least and most deprived women with breast cancer. (A) Cumulative incidence of all‐cause death and overall second primary cancer; (B) cumulative incidence of cancer of respiratory and intrathoracic organs, digestive organs, and female genital organs which have shown the most deprived quintile had a higher risk than the least deprived; (C) cumulative incidence of breast cancer and other cancers, which have shown the most deprived quintile had a lower risk than the least deprived.

Figure 1 shows the non‐parametric cumulative incidences of overall and specific SPC, accounting for all‐cause death as a competing event, and Table S3 the number of events and people at risk at different time points. The risk of overall SPC was always higher for women from the most deprived quintile compared to the least deprived quintile during the whole follow‐up period (Figure 1A). In line with data from crude rates, we observed a higher non‐parametric cumulative incidence of many types of SPC in the most than least deprived quintile, and the largest difference was for respiratory and intrathoracic organs, followed by digestive organs and female genital organs (Figure 1B). However, there was no difference in the incidence of second breast cancer during the first 10 years follow‐up, but the most deprived seemed to have a lower risk thereafter and they also had a lower risk of other types of SPC during the whole follow‐up period (Figures 1C and S2). Figure S2 shows cumulative incidence by deprivation for four specific cancers which has screening programmes within NHS England, and there were wide deprivation gaps in lung cancer incidence as SPC but not colorectal, breast or cervical cancer.

### 3.3 Relative and absolute differences in overall SPC incidence by income deprivation

**FIGURE 2:**
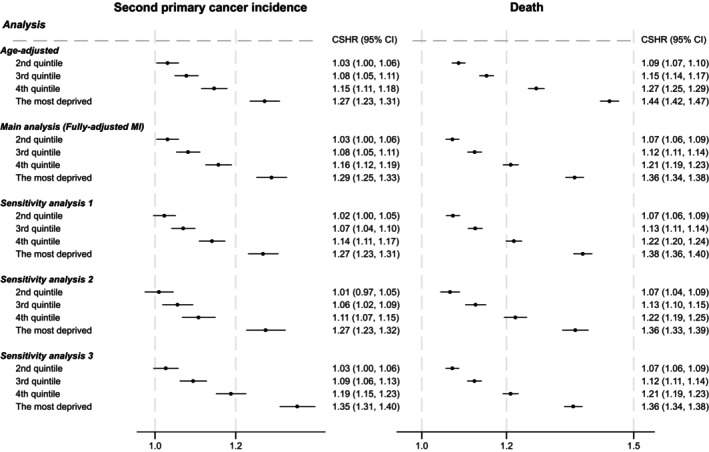
Cause‐specific hazard ratios (CSHRs) of second primary cancer incidence and death associated with income deprivation quintiles. HR, hazard ratio; MI, multiple imputation. The least deprived quintile was set as reference group. Age‐adjusted: Models only included deprivation and restricted cubic spline transformed age (*N* = 649,905). Fully adjusted MI: Models included deprivation and restricted cubic spline transformed age, ethnicity, year of primary breast cancer diagnosis group, comorbidity, primary breast cancer stage, surgery, chemotherapy, radiotherapy, and hormone therapy, and we multiply imputed missing data on ethnicity and stage (*N* = 649,905). Sensitivity analysis 1: Models were same as fully adjusted MI, but the analysis was restricted to women with complete data on all variables except stage, in which women missing on stage was grouped into a new category (*N* = 618,384). Sensitivity analysis 2: Models were same as fully adjusted MI, but the analysis was restricted to women with complete data on all variables (*N* = 409,521). Sensitivity analysis 3: Models were same as fully adjusted MI, but the analysis only included second primary non‐breast cancer as the primary cancer, and MI analysis was conducted (*N* = 649,905).

Figure 2 presents the CSHRs of SPC incidence and death by income deprivation quintile from both age adjusted models and fully adjusted multiple imputation models. Deprivation was consistently associated with increased rates of both SPC incidence and death, and CSHRs for both SPC incidence and death were increasing along with deprivation quintiles and similar in age and fully adjusted models. For the most compared to the least deprived quintile, the CSHR was 1.29 (95% CI: 1.25, 1.33) for SPC incidence and 1.36 (95% CI: 1.34, 1.38) for death in fully adjusted models (Figure 2). The CSHRs of other covariates are presented in Table S4.

**FIGURE 3:**
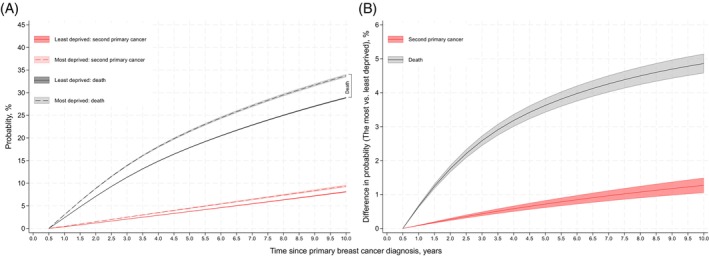
The standardised cumulative incidence of second primary cancer and death in the least and most deprived women with breast cancer. Cumulative incidence of second primary cancer and death were standardised after flexible parametric models adjusting for restricted cubic spline transformed age, ethnicity, year of primary breast cancer diagnosis group, comorbidity, primary breast cancer stage, surgery, chemotherapy, radiotherapy, and hormone therapy. Standardised cumulative incidence was computed after flexible parametric survival models, as if every woman in the least deprived and in the most deprived quintile while keeping other covariates as observed (i.e., marginal effect), and considering death as the competing event of second primary cancer incidence. Multiple imputation (10 times) was performed to account for missing data ethnicity and stage, and all estimates were combined with Rubin's rules. (A) Standardised cumulative incidence of death and overall second primary cancer in the most and least deprived quintiles. (B) Differences (the most deprived compared to the least deprived quintile) in standardised cumulative incidence of death and overall second primary cancer.

The standardised cumulative incidence (i.e., probability or risk) of death and SPC was greater for women from the most deprived compared to the least deprived quintile for the whole study period (Figure 3A). At 5 years after PBC diagnosis, the probability of getting a SPC for a woman from the least deprived income quintile was 3.7% (95% CI: 3.7, 3.8) compared to 4.5% (95% CI: 4.3, 4.6) for a woman from the most deprived income quintile, while at 10 years after PBC diagnosis, corresponding probabilities were 8.1% (95% CI: 8.0, 8.2) and 9.4% (95% CI: 9.1, 9.6) (Figure 3A and Table S5). The absolute difference (the most vs. least deprived) in the probability of being diagnosed with a SPC increased over time (Figure 3B), and differences at 5 and 10 years were 0.7% (95% CI: 0.6, 0.8) and 1.3% (95% CI: 1.0, 1.5), respectively (Table S5). The patterns for death were similar to SPC incidence, except we observed a larger standardised cumulative incidence in both the least and most deprived groups as well as a greater absolute difference between them, that is, 4.9% (95% CI: 4.6, 5.1) at 10 years after PBC diagnosis.

### 3.4 Sensitivity and stratified analyses

Figure 2 also shows the CSHRs of SPC incidence and death for three sets of sensitivity analyses, first by excluding those who had missing data on ethnicity and classifying those who had missing data on stage into a new category, second by excluding those who had missing data on ethnicity or stage, and third by only including second primary non‐breast cancer as the primary outcome. Overall, we observed very similar HRs, except a slightly stronger effect of deprivation on SPC (non‐breast cancer) incidence (analysis 3) and on death in complete‐case analysis (sensitivity analysis 2). Figure S3 presents the standardised cumulative incidence of SPC and death from these three analyses, and patterns are almost identical to those from the main analysis of multiple imputation. The absolute difference by deprivation in standardised cumulative incidence of SPC (non‐breast cancer) was 1.3% (95% CI: 1.1, 1.5) at 10 years.

Stratified analyses by year of PBC diagnosis before or after 2012 and by age at PBC diagnosis younger or older than 55 years as the proxy of menopause are given in Table S6. CSHRs before and after 2012 were similar for SPC incidence, but we observed slightly wider inequalities in death for women diagnosed with PBC after 2012 than those before (e.g., the most vs. the least deprived: 1.40 [95% CI: 1.35, 1.46] after 2012 vs. 1.35 [95% CI: 1.33, 1.38] before 2012). Conversely, we observed similar risks of death associated with deprivation stratified by age but slightly wider inequalities in SPC incidence in premenopausal women (<55 years) than postmenopausal women (≥55 years) (the most vs. the least deprived: 1.34 [95% CI: 1.26, 1.42] in premenopausal women vs. 1.27 [95% CI: 1.23, 1.32]) in postmenopausal women. Table S7 presents the HRs of income deprivation and all other covariates for both SPC incidence and death in the sensitivity analyses by censoring women at different years after the index date, and we observed similar patterns of HRs for income deprivation, except slightly wider inequalities in SPC incidence in shorter than longer follow‐up years (e.g., the most vs. least deprived, CSHR: 1.19; 95% CI: 1.13, 1.26) censoring at 1 year vs. 1.29; 1.22, 1.36) censoring at 15 years and vice versa for death ([1.41; 1.37, 1.44] vs. [1.36; 1.33, 1.39]), suggesting potential calendar and duration effects.

## 4 DISCUSSION

Using data from NCRAS between 2000 and 2018 in England, we found strong evidence of consistent and gradient socio‐economic inequalities in SPC incidence and death in women with a first PBC diagnosis. After adjusting for other sociodemographic, clinical, and tumour factors, women from the most deprived areas had about 30% higher SPC incidence and death compared to those from the least deprived areas, and these translated into an absolute risk difference of 1.3% for SPC incidence and 4.9% for death at 10 years after their PBC diagnosis. Results were consistent in sensitivity analyses by excluding those with missing data on stage and/or ethnicity and stratified analyses by year and age of diagnosis.

To our knowledge, this is the first study investigating socio‐economic inequalities in SPC incidence in women with PBC. Previous studies have mostly demonstrated the increased risk of SPC among women with breast cancer compared to the general population—the most recent meta‐analysis reported a pooled standardised incidence ratio of 1.24 (95% CI 1.14–1.36)^13^—no studies have compared the SPC incidence by deprivation. However, data from Surveillance, Epidemiology, and End Results (SEER), US cancer registries, indicated other types of inequalities—women of Hispanic or Black ethnicity had a higher risk of SPC compared to those of White ethnicity.^25^, ^26^ Two studies suggested that lower education level was associated with increased risks of SPC, consistent with our findings.^27^, ^28^

Differences in population selection and methodologies make it difficult to directly compare our findings to previous studies. In our study, 10‐year standardised cumulative incidences of SPC (i.e., probability) were 8.1% and 9.4% for women from the least and most deprived areas. Using a competing risks approach, it ranged from 6.2% to 10.5% when stratified by age groups and calendar year of diagnosis in data from 11 French cancer registries,^29^ whereas in United States, it was 4.4% for second PBC and 7.8% for second primary non‐breast cancer in SEER data,^30^ and 10.8% for any SPC in the Kaiser Permanente Cohort.^6^ However, our crude SPC incidence rate was slightly higher than in another study using SEER data—11.1 versus 9.5 per 1000 person‐years.^31^ It should be noted that Kaiser cohort included slightly younger women (median age: 60.7 vs. 62.0 years in our study),^6^ and one study using SEER data only included women aged between 20 and 80 years old.^30^ In addition, the start of observations for risk of SPC varied substantially, from 61 days after PBC diagnosis in the French study^29^ to 5 years in SEER data study.^30^ We included different calendar year of PBC diagnosis in these studies, for example, SEER data diagnosed between 2002 and 2011 versus between 2000 and 2018 in our study, and SPC incidence rate might be increasing by calendar year due to improved survival.^32^

We also observed similarities and differences in the SPC incidence of specific cancers. The most common SPCs in our cohort were cancer of digestive organs followed by breast cancer and respiratory and intrathoracic organs, particularly among women from deprived areas. Similar to our data, the most common SPC was colorectal cancer in French cancer registries,^29^ and there was a bi‐directional relationship between breast and corpus uteri cancer.^33^ While in the Kaiser Permanent Cohort, there were more contralateral breast, followed by lung and bronchus, and colon cancer.^6^ The different sociodemographic and tumour characteristics of included women, for example, age distribution, ethnicity, and tumour factors, and public health programmes and contextual healthcare systems across different countries might also contribute to these diverse findings.

This study has some strengths and limitations. First, we have used population‐based cancer registries from England, which covers 99% of people with cancer and data are of high quality.^17^ However, there were still some missing data in registries, particularly tumour factors in earlier years of the study, and the missing proportion of stage reduced to about 10% after 2012. We have conducted multiple imputation to handle the missing data and our results were robust across sensitivity analyses, including stratified by calendar year. We did not include other tumour factors such as ER, PR or HER2 status due to very high missing proportions. Future studies with more complete data should investigate their roles in socio‐economic inequalities in SPC. Second, some variables in our study relied on complete and accurate clinical coding, and since these data were not collected for research purpose, we cannot rule out misclassification. However, we do not expect misclassifications would differ by income deprivation and therefore should not affect our findings. In particular, the definition of SPC outcome—how to define a second PBC—has an impact on risk estimation.^24^ We have estimated crude 10‐year cumulative risk based on different definitions (Table S8) and also conducted a sensitivity analysis by only including non‐breast cancer only as the primary outcome for SPC, and we found similar deprivation gaps across different definitions. Third, due to lack of data on individual socio‐economic status, we used small area‐based income deprivation as a proxy which may not fully reflect the individual's income.^34^ The role of individual (i.e., person) versus area‐based (i.e., place) characteristics may differ with respect to cancer outcomes as they characterise different aspects of risk.^35^, ^36^ Future health data research should make an effort to make both measures available for researchers. Fourth, inequalities in breast cancer survival exist may influence the observed risk of SPC. We modelled both death and SPC incidence and considered death as the competing risk to minimise this impact. Lastly, although we adjusted for many potential confounders including other sociodemographic, clinical, and tumour factors, some unmeasured confounding may remain.

Incidence of breast cancer was higher in women (general population) from less deprived areas in England,^15^ incidence of SPC was however higher in women from more deprived areas in our study. Our findings on increased risks of SPC and death associated with deprivation were in line with associations between deprivation and all cancer incidence and premature death in the general population,^37^, ^38^ though with a smaller magnitude. One of the possible explanations is that variations in risk factors (e.g., age, lifestyle, etc.) by deprivation are larger in the general population than in women diagnosed with breast cancer. Further research with such data in both general population and women with breast cancer is warranted to investigate this. Some cancer treatments that could increase the risk of SPC,^3^ such as radiotherapy, were more commonly prescribed to women from less deprived areas in our study. However, in adjusted models, we still observed higher risk of SPC in women from more deprived areas and adjusting for receipt of cancer treatments did not explain the inequalities in SPC incidence. Of note, in our main models, we have adjusted for prevalent treatments only (i.e., initiated within 6 months after diagnosis), and many women may still be on treatments up to 1 year after diagnosis, but our analyses showed no differences in deprivation gaps (not reported), regardless of time windows used to determine treatment status. Lifestyle factors such as alcohol assumption, smoking, and body weight have also been associated with SPC incidence.^39^, ^40^ We were unable to account for differences in lifestyle factors since these were not routinely collected in the English cancer registry. Furthermore, primary cancer diagnosis is considered as the ‘teachable moment’ for many patients,^41^ but changes in lifestyles or capacities to make changes may not be proportional by socio‐economic status.^42^ In addition, familial risks and genetic predisposition were also shown to be related to SPC incidence,^28^, ^43^, ^44^ and these might be more common in some ethnic groups than others, and ethnicity is high correlated with socio‐economic status,^45^ though we have adjusted for ethnicity in our study. Lastly, similar to potential mechanisms for inequalities in survival and recurrence, access to healthcare facilities, optimal treatments for primary cancer, and post‐treatment follow‐ups may also contribute to observed inequalities.

Our findings have clinical and public health implications. The findings of income deprivation as a potential risk factor for SPC incidence corroborates the conclusions drawn from earlier research that high socio‐economic status is associated with lower recurrence risk and better prognosis after recurrence.^46^ This reaffirms the well‐established understanding that socio‐economic factors significantly contribute to health disparities. Based on our findings, of every 55,000 new breast cancer cases every year, we could have reduced more than 600 cases of SPC at 10 years after diagnosis if inequalities were eliminated, and this figure might increase given the improved survival in recent years. Indeed, we observed some calendar effects in our study, and future study should quantify the temporal trends of SPC incidence in this population to inform future care needs.

In conclusion, women diagnosed with PBC from deprived areas in England faced a substantially higher risk of SPC than those from less deprived areas. Expanding existing cancer screening programmes, such as lung health checks,^47^ to women with breast cancer, particularly in those from deprived areas, may help to identify SPC at an earlier stage to minimise disparities. Moving forward, future research should also explore the mechanisms underlying these disparities, including the contribution of known risk factors, such as tobacco smoking, access to healthcare, health behaviours, and environmental exposures. Identifying factors that contribute to inequalities in SPC incidence after PBC is crucial for informing targeted interventions aimed at mitigating health inequalities among breast cancer survivors.

## AUTHOR CONTRIBUTIONS

**Ruchika Golani:** Conceptualization; writing – review and editing; writing – original draft; data curation; investigation. **Eva Kagenaar:** Visualization; writing – original draft; writing – review and editing. **Jérémie Jégu:** Validation; writing – review and editing. **Aurélien Belot:** Conceptualization; methodology; supervision; writing – review and editing. **Suping Ling:** Conceptualization; methodology; data curation; formal analysis; supervision; writing – original draft; writing – review and editing; visualization.

## FUNDING INFORMATION

S.L. and A.B., as a member of Inequalities in Cancer Outcome Network group, are funded by Cancer Research UK programme (Grant No. C7923/A29018). E.K. is funded by the National Institute for Health and Care Research (Grant No. NIHR153580). The funders have no role in study design, data collection, data analysis, interpretation, or writing of the article.

## CONFLICT OF INTEREST STATEMENT

All authors declare no conflicts of interest.

## ETHICS STATEMENT

The use of data has been approved by NHS Health Research Authority London – Central Research Ethics Committee (REC reference: 21/LO/0552; IRAS project ID: 279592) and this study protocol by LSHTM Ethics Online (reference: 29150). Current legislation (GDPR and the DPA 2018) makes it permissible to use individual and even sensitive personal data, without consent, for bona fide non‐interventional public health research, provided the relevant statutory and ethical permissions have been acquired from HRA and An NHS Research Ethics Committee, respectively. The wishes of patients who have withheld or withdrawn their consent are respected for identifiable data by the data providers (NHS and PHE). Data received by Inequalities in Cancer Outcome Network group have been anonymised.

## Supporting information


Data S1.

## Data Availability

Data access is permitted via authorisation from NHS digital only. Statistical codes for data processing and analyses are available at GitHub (https://github.com/supingling/spc). Further information is available from the corresponding author upon request.
