# Supplementary material for: Socio‐economic inequalities in second primary cancer incidence: A competing risks analysis of women with breast cancer in England between 2000 and 2018
Source: Int J Cancer. 2025 Jan 24;156(12):2283–93. doi: 10.1002/ijc.35320 (PMC12008825; doi:10.1002/ijc.35320)
Supplement: Supplementary file 1 — Data S1. [file IJC-156-2283-s001.pdf]

Supplemental material for

Socioeconomic inequalities in second primary cancer incidence: a competing risks analysis of women with breast cancer in England between 2000 – 2018

Ruchika Golani, Eva Kagenaar, Jérémie Jégu, Aurélien Belot, Suping Ling

Contents

Table S1. Baseline characteristics on the day of primary breast cancer diagnosis between patients with missing and complete data for ethnicity and ethnicity and primary breast cancer (PBC) stage. 2

Table S2. The number of events and crude rates for SPC and death in the 2nd, 3rd and 4th quintile of income 4

Table S3. The number of events and people at risk for SPC and death in the least and most deprived quintiles 5

Table S4. Hazard ratios for second primary cancer incidence and death for other adjusted covariates in the main analysis and two sensitivity analyses. 6

Table S5. Probability (%) and 95% Confidence Interval of second primary cancer incidence and death for the most and least deprived income quintile. 7

Table S6. Hazard ratios for second primary cancer and death in stratified analyses by year and age of diagnosis 8

Table S7. Hazard ratios and 95% Confidence Interval for second primary cancers and death in sensitivity analyses by censoring women at different years after the index date. 9

Table S8. 10-year cumulative risk of overall second primary cancer (SPC) and second primary breast cancer by different definitions 10

Figure S1. Flowchart of participants selection 11

Figure S2. The non-parametric cumulative incidence of second primary cancer of selected sites in the least and most deprived women with breast cancer 12

Figure S3. The standardised cumulative incidence of second primary cancer incidence and death for models including women with complete data on ethnicity and women with complete data on ethnicity and breast cancer stage. 13

Table S1. Baseline characteristics on the day of primary breast cancer diagnosis between patients with missing and complete data for ethnicity and ethnicity and primary breast cancer (PBC) stage.

	Ethnicity		Ethnicity and PBC stage		
	**Complete cases**	**Missing**	**Complete cases**	**Missing**	**Total**
	**N=618,384**	**N=31,521**	**N=409,521**	**N=240,384**	**N=649,905**
Year of diagnosis					
2000-2003	117,610 (19.0%)	10,642 (33.8%)	59,948 (14.6%)	68,304 (28.4%)	128,252 (19.7%)
2004-2007	130,932 (21.2%)	5,000 (15.9%)	60,986 (14.9%)	74,946 (31.2%)	135,932 (20.9%)
2008-2011	137,393 (22.2%)	3,286 (10.4%)	75,197 (18.4%)	65,482 (27.2%)	140,679 (21.6%)
2012-2014	107,525 (17.4%)	4,635 (14.7%)	94,915 (23.2%)	17,245 (7.2%)	112,160 (17.3%)
2015-2018	124,924 (20.2%)	7,958 (25.2%)	118,475 (28.9%)	14,407 (6.0%)	132,882 (20.4%)
Age at diagnosis, years	62.0 (51.5-72.7)	62.9 (52.1-76.7)	61.6 (51.4-71.2)	62.8 (51.7-75.9)	62.0 (51.5-72.9)
Age at diagnosis group, years					
18.0-44.9	68,494 (11.1%)	2,888 (9.2%)	44,000 (10.7%)	27,382 (11.4%)	71,382 (11.0%)
45.0-54.9	136,584 (22.1%)	7,243 (23.0%)	93,938 (22.9%)	49,889 (20.8%)	143,827 (22.1%)
55.0-64.9	151,266 (24.5%)	7,094 (22.5%)	103,757 (25.3%)	54,603 (22.7%)	158,360 (24.4%)
65.0-74.9	131,735 (21.3%)	5,681 (18.0%)	92,658 (22.6%)	44,758 (18.6%)	137,416 (21.1%)
75.0-99.9	130,305 (21.1%)	8,615 (27.3%)	75,168 (18.4%)	63,752 (26.5%)	138,920 (21.4%)
Ethnicity					
White	580,498 (93.9%)	0 (0.0%)	385,548 (94.1%)	194,950 (81.1%)	580,498 (89.3%)
Asian	18,083 (2.9%)	0 (0.0%)	11,908 (2.9%)	6,175 (2.6%)	18,083 (2.8%)
Black	10,547 (1.7%)	0 (0.0%)	6,221 (1.5%)	4,326 (1.8%)	10,547 (1.6%)
Other	9,256 (1.5%)	0 (0.0%)	5,844 (1.4%)	3,412 (1.4%)	9,256 (1.4%)
Missing	0 (0.0%)	31,521 (100.0%)	0 (0.0%)	31,521 (13.1%)	31,521 (4.9%)
Stage					
I	112,887 (18.3%)	4,732 (15.0%)	112,887 (27.6%)	4,732 (2.0%)	117,619 (18.1%)
II	237,727 (38.4%)	10,403 (33.0%)	237,727 (58.1%)	10,403 (4.3%)	248,130 (38.2%)
III	40,048 (6.5%)	1,521 (4.8%)	40,048 (9.8%)	1,521 (0.6%)	41,569 (6.4%)
IV	18,859 (3.0%)	1,038 (3.3%)	18,859 (4.6%)	1,038 (0.4%)	19,897 (3.1%)
Missing	208,863 (33.8%)	13,827 (43.9%)	0 (0.0%)	222,690 (92.6%)	222,690 (34.3%)
Comorbidity	21,268 (3.4%)	272 (0.9%)	14,576 (3.6%)	6,964 (2.9%)	21,540 (3.3%)
Treatment overview					
Curative surgery only	128,992 (20.9%)	6,887 (21.8%)	76,232 (18.6%)	59,647 (24.8%)	135,879 (20.9%)
Curative surgery with radiotherapy	180,941 (29.3%)	7,246 (23.0%)	140,686 (34.4%)	47,501 (19.8%)	188,187 (29.0%)
Curative surgery with chemotherapy	130,989 (21.2%)	3,976 (12.6%)	92,485 (22.6%)	42,480 (17.7%)	134,965 (20.8%)
Curative surgery with radiotherapy and chemotherapy	20,407 (3.3%)	785 (2.5%)	14,026 (3.4%)	7,166 (3.0%)	21,192 (3.3%)

Radiotherapy only	18,576 (3.0%)	1,503 (4.8%)	11,514 (2.8%)	8,565 (3.6%)	20,079 (3.1%)
Chemotherapy only	35,985 (5.8%)	1,587 (5.0%)	24,088 (5.9%)	13,484 (5.6%)	37,572 (5.8%)
Radiotherapy and chemotherapy	6,631 (1.1%)	320 (1.0%)	4,759 (1.2%)	2,192 (0.9%)	6,951 (1.1%)
No cancer-directed treatment	95,863 (15.5%)	9,217 (29.2%)	45,731 (11.2%)	59,349 (24.7%)	105,080 (16.2%)
Curative surgery					
No	157,055 (25.4%)	12,627 (40.1%)	86,092 (21.0%)	83,590 (34.8%)	169,682 (26.1%)
Yes	461,329 (74.6%)	18,894 (59.9%)	323,429 (79.0%)	156,794 (65.2%)	480,223 (73.9%)
Radiotherapy					
No	391,829 (63.4%)	21,667 (68.7%)	238,536 (58.2%)	174,960 (72.8%)	413,496 (63.6%)
Yes	226,555 (36.6%)	9,854 (31.3%)	170,985 (41.8%)	65,424 (27.2%)	236,409 (36.4%)
Chemotherapy					
No	424,372 (68.6%)	24,853 (78.8%)	274,163 (66.9%)	175,062 (72.8%)	449,225 (69.1%)
Yes	194,012 (31.4%)	6,668 (21.2%)	135,358 (33.1%)	65,322 (27.2%)	200,680 (30.9%)
Hormone therapy					
No	392,060 (63.4%)	20,275 (64.3%)	247,765 (60.5%)	164,570 (68.5%)	412,335 (63.4%)
Yes	226,324 (36.6%)	11,246 (35.7%)	161,756 (39.5%)	75,814 (31.5%)	237,570 (36.6%)

Table S2. The number of events and crude rates for SPC and death in the 2nd, 3rd and 4th quintile of income

	**2nd quintile (Person-years: 985,031)**			**3rd quintile (Person-years: 886,677)**			**4th quintile (Person-years: 755,346)**	
	Events	Incidence rate		Events	Incidence rate		Events	Incidence rate
Overall second primary cancer	10,463	10.62 (10.42, 10.83)		9,834	11.09 (10.87, 11.31)		8,826	11.68 (11.44, 11.93)
Second breast cancer (C50)	2,211	2.24 (2.15, 2.34)		1,948	2.20 (2.10, 2.30)		1,662	2.20 (2.10, 2.31)
Second cancer female genital organs (C51-C58)	1,677	1.70 (1.62, 1.79)		1,518	1.71 (1.63, 1.80)		1,415	1.87 (1.78, 1.97)
Second cancer of digestive organs (C15-C26)	2,498	2.54 (2.44, 2.64)		2,350	2.65 (2.55, 2.76)		2,086	2.76 (2.65, 2.88)
Second cancer of respiratory and intrathoracic organs (C30-C39)	1,299	1.32 (1.25, 1.39)		1,420	1.60 (1.52, 1.69)		1,492	1.98 (1.88, 2.08)
Other second cancers	2,778	2.82 (2.72, 2.93)		2,598	2.93 (2.82, 3.04)		2,171	2.87 (2.76, 3.00)
Death	36,670	37.23 (36.85, 37.61)		36,011	40.61 (40.20, 41.04)		34,211	45.29 (44.81, 45.77)

Table S3. The number of events and people at risk for SPC and death in the least and most deprived quintiles

Years since primary breast cancer diagnosis	0.5	1	2	3	4	5	6	7	8	9	10	11	12	13	14	15	16	17	18
The least deprived																			
Number at risk	149,720	141,912	127,048	113,092	100,269	88,643	78,120	68,850	60,160	52,243	44,797	38,002	31,626	25,914	20,517	15,574	10,765	6,755	3,229
Overall SPC	504	1,119	1,026	979	927	855	735	710	668	621	502	481	353	286	255	203	153	75	25
Second breast cancer	84	203	204	182	194	173	138	145	146	148	118	135	87	58	69	56	47	21	6
Second cancer of female genital organs	87	160	190	140	136	142	119	117	100	103	71	65	54	50	48	23	19	6	3
Second cancer of digestive organs	128	277	251	262	234	207	174	168	162	141	136	108	80	89	46	46	40	15	8
Second cancer of respiratory and intrathoracic organs	49	121	103	117	112	90	98	75	72	69	49	61	37	26	30	23	14	15	2
Other second cancers	156	358	278	278	251	243	206	205	188	160	128	112	95	63	62	55	33	18	6
Death	2,650	5,468	4,791	4,021	3,139	2,518	2,102	1,746	1,451	1,181	1,023	896	697	616	442	330	209	114	39
The most deprived																			
Number at risk	99,092	92,769	80,766	70,263	61,117	53,099	46,101	39,897	34,258	29,323	24,783	20,638	17,025	13,692	10,617	7,881	5,520	3,396	1,602
Overall SPC	409	845	825	730	679	603	580	568	475	447	383	303	254	204	185	139	101	47	22
Second breast cancer	35	138	133	134	126	114	97	86	107	91	63	72	35	47	35	23	19	9	4
Second cancer of female genital organs	65	127	113	82	111	87	88	93	83	63	40	48	40	26	30	21	13	6	3
Second cancer of digestive organs	100	218	186	193	148	134	134	135	90	86	103	55	57	54	43	32	26	3	3
Second cancer of respiratory and intrathoracic organs	104	170	169	162	155	137	128	138	92	104	91	68	63	40	35	36	26	18	9
Other second cancers	105	192	224	159	139	131	133	116	103	103	86	60	59	37	42	27	17	11	3
Death	2,972	5,727	4,703	3,683	2,839	2,230	1,852	1,425	1,173	964	809	701	535	438	334	236	162	93	22

SPC: second primary cancer.

Should be interpreted together with Figure 1.

Table S4. Hazard ratios for second primary cancer incidence and death for other adjusted covariates in the main analysis and two sensitivity analyses.

	Hazard Ratio (95% confidence interval)							
	**Fully adjusted MI**		**Fully adjusted sensitivity analysis 1**		**Fully adjusted sensitivity analysis 2**		**Fully adjusted sensitivity analysis 3**	
	**SPC incidence**	**Death**	**SPC incidence**	**Death**	**SPC incidence**	**Death**	**SPC incidence**	**Death**
Ethnicity [a]								
Asian	0.71 (0.67, 0.77)	0.91 (0.88, 0.94)	0.71 (0.67, 0.77)	0.89 (0.86, 0.92)	0.71 (0.64, 0.78)	0.91 (0.86, 0.95)	0.70 (0.65, 0.76)	0.91 (0.88, 0.94)
Black	0.84 (0.77, 0.92)	1.16 (1.11, 1.21)	0.85 (0.78, 0.93)	1.15 (1.10, 1.20)	0.85 (0.74, 0.97)	1.14 (1.07, 1.22)	0.79 (0.71, 0.87)	1.16 (1.11, 1.21)
Other	0.76 (0.69, 0.84)	0.93 (0.88, 0.97)	0.77 (0.69, 0.85)	0.91 (0.87, 0.96)	0.79 (0.68, 0.91)	0.91 (0.84, 0.98)	0.75 (0.67, 0.84)	0.94 (0.89, 0.98)
Comorbidity [a]	1.09 (1.05, 1.13)	1.27 (1.26, 1.28)	1.16 (1.09, 1.22)	1.63 (1.59, 1.66)	1.20 (1.12, 1.29)	1.64 (1.59, 1.69)	1.02 (0.99, 1.05)	1.52 (1.50, 1.54)
Chemotherapy [a]	1.03 (1.00, 1.05)	1.52 (1.50, 1.54)	1.01 (0.98, 1.03)	1.52 (1.50, 1.54)	0.99 (0.96, 1.03)	1.44 (1.41, 1.47)	1.17 (1.10, 1.24)	1.58 (1.54, 1.61)
Curative surgery [a]	1.02 (1.00, 1.05)	0.57 (0.56, 0.57)	1.01 (0.99, 1.03)	0.59 (0.58, 0.59)	0.98 (0.95, 1.01)	0.65 (0.64, 0.66)	1.05 (1.02, 1.08)	0.57 (0.56, 0.57)
Hormone therapy [a]	0.99 (0.97, 1.01)	0.98 (0.97, 0.99)	0.97 (0.95, 0.99)	0.99 (0.98, 1.00)	0.98 (0.95, 1.00)	0.98 (0.96, 0.99)	1.00 (0.98, 1.02)	0.99 (0.97, 1.00)
Radiotherapy [a]	0.99 (0.97, 1.01)	0.82 (0.81, 0.83)	0.98 (0.96, 1.00)	0.83 (0.82, 0.84)	0.99 (0.96, 1.01)	0.83 (0.81, 0.84)	0.97 (0.95, 1.00)	0.82 (0.81, 0.83)
Breast cancer stage [a]								
II	0.99 (0.97, 1.02)	1.27 (1.25, 1.29)	1.00 (0.97, 1.03)	1.50 (1.47, 1.53)	1.01 (0.98, 1.03)	1.52 (1.50, 1.55)	1.00 (0.97, 1.03)	1.27 (1.25, 1.29)
III	1.06 (1.01, 1.11)	1.83 (1.79, 1.87)	1.09 (1.03, 1.14)	2.92 (2.85, 2.99)	1.09 (1.04, 1.15)	3.08 (3.01, 3.16)	1.00 (0.95, 1.06)	1.82 (1.79, 1.86)
IV	0.95 (0.89, 1.02)	2.96 (2.88, 3.04)	0.92 (0.85, 0.99)	6.08 (5.94, 6.24)	0.91 (0.84, 0.99)	6.57 (6.40, 6.73)	0.95 (0.88, 1.03)	2.94 (2.87, 3.02)
Missing	-	-	0.93 (0.91, 0.95)	1.81 (1.78, 1.84)	-	-		
Year of diagnosis [a]								
2004-2007	1.03 (1.00, 1.05)	0.86 (0.85, 0.87)	1.03 (1.01, 1.06)	0.91 (0.90, 0.92)	1.03 (0.99, 1.06)	0.88 (0.87, 0.90)	1.02 (0.99, 1.04)	0.86 (0.85, 0.87)
2008-2011	1.06 (1.03, 1.09)	0.72 (0.71, 0.73)	1.07 (1.04, 1.10)	0.80 (0.79, 0.82)	1.06 (1.02, 1.10)	0.76 (0.74, 0.78)	1.06 (1.03, 1.09)	0.73 (0.72, 0.74)
2012-2014	1.08 (1.04, 1.12)	0.66 (0.65, 0.67)	1.08 (1.04, 1.12)	0.76 (0.75, 0.78)	1.06 (1.02, 1.11)	0.72 (0.71, 0.74)	1.09 (1.05, 1.13)	0.66 (0.65, 0.67)
2015-2018	1.06 (1.00, 1.11)	0.61 (0.59, 0.62)	1.08 (1.02, 1.13)	0.72 (0.70, 0.74)	1.05 (0.99, 1.12)	0.70 (0.68, 0.72)	1.05 (0.99, 1.11)	0.61 (0.60, 0.63)

[a] Reference category are Ethnicity: White, Comorbidity: No' Chemotherapy: No, Curative surgery: No, Hormone therapy: No, Radiotherapy: No, Breast cancer stage: I.

SPC: second primary cancer Fully adjusted MI: Multiple imputation. Fully adjusted sensitivity analysis 1: excluding women with missing data on ethnicity and grouping missing stage as a new category. Fully adjusted sensitivity analysis 2: complete case analysis by excluding women with missing data on ethnicity and primary breast cancer stage. Fully adjusted sensitivity analysis 3: the primary outcome only included second primary non-breast cancer (MI analysis).

Table S5. Probability (%) and 95% Confidence Interval of second primary cancer incidence and death for the most and least deprived income quintile.

	Probability (95% confidence interval)					
	Second primary cancer incidence			Death		
Years since PBC diagnosis	The least deprived	The most deprived	Difference	The least deprived	The most deprived	Difference
1	0.4 (0.4, 0.4)	0.5 (0.5, 0.5)	0.1 (0.1, 0.1)	2.4 (2.3, 2.4)	3.0 (3.0, 3.1)	0.7 (0.6, 0.7)
3	2.1 (2.0, 2.1)	2.5 (2.4, 2.6)	0.4 (0.4, 0.5)	11.3 (11.2, 11.4)	13.9 (13.7, 14.1)	2.6 (2.4, 2.7)
5	3.7 (3.7, 3.8)	4.5 (4.3, 4.6)	0.7 (0.6, 0.8)	17.9 (17.8, 17.9)	21.5 (21.3, 21.7)	3.6 (3.4, 3.8)
10	8.1 (8.0, 8.2)	9.4 (9.1, 9.6)	1.3 (1.0, 1.5)	28.9 (28.8, 29.0)	33.7 (33.4, 34.1)	4.9 (4.6, 5.1)

Fully adjusted multiple imputation models. Standardised cumulative incidence was computed after flexible parametric survival models, as if every woman in the least deprived and in the most deprived quintile while keeping other covariates as observed (i.e., marginal effect), and considering death as the competing event of second primary cancer incidence. Difference was between the most and least deprived.

Table S6. Hazard ratios for second primary cancer and death in stratified analyses by year and age of diagnosis

	Hazard Ratio (95% confidence interval)							
	Before 2012		After 2012		<55 years		≥ 55 years	
	SPC	Death	SPC	Death	SPC	Death	SPC	Death
**Age adjusted**								
2nd quintile	1.04 (1.01, 1.07)	1.08 (1.06, 1.09)	1.01 (0.94, 1.08)	1.12 (1.08, 1.17)	1.06 (1.00, 1.12)	1.07 (1.04, 1.11)	1.02 (0.99, 1.05)	1.09 (1.07, 1.11)
3rd quintile	1.08 (1.05, 1.11)	1.14 (1.13, 1.16)	1.08 (1.00, 1.16)	1.18 (1.14, 1.22)	1.09 (1.03, 1.15)	1.15 (1.11, 1.19)	1.07 (1.04, 1.11)	1.15 (1.13, 1.17)
4th quintile	1.16 (1.13, 1.20)	1.25 (1.23, 1.27)	1.07 (0.99, 1.16)	1.35 (1.30, 1.40)	1.18 (1.11, 1.25)	1.28 (1.24, 1.32)	1.14 (1.10, 1.17)	1.27 (1.24, 1.29)
Most deprived	1.27 (1.23, 1.31)	1.41 (1.39, 1.44)	1.28 (1.18, 1.38)	1.55 (1.49, 1.61)	1.33 (1.25, 1.41)	1.42 (1.37, 1.47)	1.25 (1.21, 1.30)	1.45 (1.42, 1.47)
**Fully adjusted MI**								
2nd quintile	1.04 (1.01, 1.07)	1.07 (1.05, 1.08)	1.01 (0.94, 1.08)	1.12 (1.08, 1.16)	1.06 (1.00, 1.12)	1.06 (1.03, 1.10)	1.02 (0.99, 1.05)	1.07 (1.05, 1.09)
3rd quintile	1.08 (1.05, 1.12)	1.12 (1.10, 1.14)	1.08 (1.01, 1.16)	1.15 (1.11, 1.19)	1.09 (1.03, 1.15)	1.12 (1.09, 1.16)	1.08 (1.05, 1.11)	1.12 (1.10, 1.14)
4th quintile	1.17 (1.14, 1.21)	1.20 (1.18, 1.22)	1.08 (1.00, 1.17)	1.26 (1.21, 1.31)	1.18 (1.12, 1.26)	1.22 (1.18, 1.26)	1.15 (1.11, 1.19)	1.20 (1.18, 1.22)
Most deprived	1.29 (1.25, 1.33)	1.35 (1.33, 1.38)	1.29 (1.19, 1.39)	1.40 (1.35, 1.46)	1.34 (1.26, 1.42)	1.35 (1.30, 1.40)	1.27 (1.23, 1.32)	1.36 (1.33, 1.38)

SPC: second primary cancer. Reference group is least deprived quintile. Age adjusted: models only included age and income deprivation. Fully adjusted MI: models included all covariates and multiply imputed 10 time as the main analysis.

Table S7. Hazard ratios and 95% Confidence Interval for second primary cancers and death in sensitivity analyses by censoring women at different years after the index date.

	Censoring at different years after the index date, Hazard Ratio (95% confidence interval)				
	1 year	3 years	5 years	10 years	15 years
Second primary cancer					
Income quintile					
2nd quintile	1.02 (0.97, 1.07)	1.02 (0.97, 1.07)	1.01 (0.96, 1.07)	1.02 (0.97, 1.07)	1.03 (0.98, 1.09)
3rd quintile	1.03 (0.98, 1.08)	1.05 (1.00, 1.10)	1.07 (1.01, 1.12)	1.07 (1.02, 1.13)	1.09 (1.03, 1.14)
4th quintile	1.13 (1.07, 1.19)	1.12 (1.07, 1.18)	1.12 (1.07, 1.18)	1.14 (1.08, 1.20)	1.16 (1.10, 1.22)
Most deprived	1.19 (1.13, 1.26)	1.23 (1.16, 1.30)	1.22 (1.15, 1.28)	1.27 (1.21, 1.34)	1.29 (1.22, 1.36)
Ethnicity [a]					
Asian	0.77 (0.68, 0.88)	0.74 (0.66, 0.84)	0.76 (0.67, 0.86)	0.72 (0.63, 0.81)	0.71 (0.62, 0.80)
Black	0.77 (0.65, 0.90)	0.81 (0.69, 0.95)	0.83 (0.71, 0.98)	0.86 (0.74, 1.02)	0.85 (0.72, 1.00)
Other	0.86 (0.72, 1.02)	0.85 (0.72, 1.01)	0.83 (0.70, 0.98)	0.79 (0.67, 0.93)	0.76 (0.64, 0.89)
Comorbidity [a]	1.06 (1.01, 1.11)	1.06 (1.01, 1.11)	1.08 (1.03, 1.13)	1.09 (1.04, 1.14)	1.09 (1.04, 1.14)
Chemotherapy [a]	0.85 (0.81, 0.89)	0.96 (0.92, 1.01)	0.99 (0.94, 1.04)	1.01 (0.96, 1.06)	1.02 (0.97, 1.08)
Curative surgery [a]	0.95 (0.92, 1.00)	1.01 (0.97, 1.05)	1.01 (0.97, 1.05)	1.03 (0.98, 1.07)	1.03 (0.99, 1.07)
Hormone therapy [a]	1.00 (0.97, 1.04)	0.98 (0.94, 1.01)	0.97 (0.94, 1.01)	0.98 (0.94, 1.01)	0.98 (0.95, 1.02)
Radiotherapy [a]	0.92 (0.88, 0.96)	0.96 (0.92, 1.00)	0.98 (0.94, 1.02)	0.99 (0.95, 1.03)	0.99 (0.95, 1.03)
Breast cancer stage [a]					
II	1.00 (0.95, 1.05)	1.01 (0.96, 1.06)	1.00 (0.95, 1.04)	0.99 (0.95, 1.04)	0.99 (0.95, 1.04)
III	1.14 (1.05, 1.24)	1.11 (1.03, 1.20)	1.10 (1.03, 1.18)	1.07 (1.00, 1.15)	1.06 (0.99, 1.14)
IV	1.09 (0.98, 1.21)	1.00 (0.91, 1.10)	0.93 (0.85, 1.03)	0.95 (0.87, 1.05)	0.95 (0.86, 1.05)
Year of diagnosis [a]					
2004-2007	1.08 (1.02, 1.13)	1.09 (1.04, 1.15)	1.07 (1.01, 1.12)	1.04 (0.99, 1.09)	1.03 (0.98, 1.08)
2008-2011	1.11 (1.05, 1.17)	1.15 (1.09, 1.21)	1.12 (1.06, 1.17)	1.07 (1.01, 1.12)	1.06 (1.01, 1.12)
2012-2014	1.21 (1.15, 1.28)	1.17 (1.11, 1.24)	1.11 (1.05, 1.17)	1.09 (1.03, 1.15)	1.08 (1.02, 1.14)
2015-2018	1.22 (1.14, 1.30)	1.13 (1.06, 1.20)	1.09 (1.03, 1.17)	1.06 (1.00, 1.13)	1.06 (0.99, 1.13)
Death					
Income quintile					
2nd quintile	1.10 (1.07, 1.12)	1.07 (1.05, 1.09)	1.08 (1.05, 1.10)	1.07 (1.05, 1.10)	1.07 (1.05, 1.10)
3rd quintile	1.15 (1.12, 1.18)	1.12 (1.10, 1.15)	1.12 (1.09, 1.15)	1.12 (1.10, 1.15)	1.12 (1.10, 1.15)
4th quintile	1.27 (1.24, 1.30)	1.21 (1.18, 1.24)	1.20 (1.18, 1.23)	1.21 (1.18, 1.24)	1.21 (1.18, 1.24)
Most deprived	1.41 (1.37, 1.44)	1.37 (1.34, 1.40)	1.36 (1.32, 1.39)	1.35 (1.32, 1.39)	1.36 (1.33, 1.39)
Ethnicity [a]					
Asian	0.97 (0.91, 1.03)	0.93 (0.88, 0.98)	0.91 (0.86, 0.96)	0.91 (0.86, 0.96)	0.91 (0.86, 0.96)
Black	1.25 (1.17, 1.33)	1.24 (1.17, 1.31)	1.22 (1.15, 1.29)	1.18 (1.11, 1.25)	1.16 (1.10, 1.23)
Other	0.94 (0.87, 1.02)	0.93 (0.86, 1.00)	0.93 (0.86, 1.00)	0.93 (0.87, 1.00)	0.92 (0.86, 0.99)
Comorbidity [a]	1.28 (1.26, 1.29)	1.27 (1.25, 1.28)	1.27 (1.26, 1.29)	1.27 (1.26, 1.29)	1.27 (1.25, 1.29)
Chemotherapy [a]	1.66 (1.62, 1.70)	1.66 (1.63, 1.70)	1.62 (1.59, 1.66)	1.56 (1.53, 1.59)	1.53 (1.50, 1.56)
Curative surgery [a]	0.36 (0.35, 0.37)	0.43 (0.42, 0.44)	0.47 (0.46, 0.48)	0.53 (0.52, 0.54)	0.56 (0.55, 0.57)
Hormone therapy [a]	0.82 (0.80, 0.83)	0.91 (0.89, 0.92)	0.94 (0.93, 0.96)	0.98 (0.96, 0.99)	0.98 (0.97, 1.00)
Radiotherapy [a]	1.00 (0.98, 1.02)	0.87 (0.85, 0.89)	0.83 (0.82, 0.85)	0.82 (0.81, 0.84)	0.82 (0.81, 0.84)
Breast cancer stage [a]					
II	1.30 (1.25, 1.35)	1.34 (1.31, 1.38)	1.33 (1.30, 1.37)	1.30 (1.26, 1.33)	1.27 (1.24, 1.30)
III	2.01 (1.94, 2.08)	2.04 (1.98, 2.11)	1.97 (1.91, 2.04)	1.88 (1.82, 1.93)	1.83 (1.78, 1.89)
IV	3.66 (3.50, 3.82)	3.48 (3.36, 3.61)	3.27 (3.17, 3.39)	3.04 (2.94, 3.14)	2.97 (2.88, 3.07)
Year of diagnosis [a]					
2004-2007	0.83 (0.82, 0.85)	0.86 (0.84, 0.87)	0.85 (0.84, 0.87)	0.86 (0.84, 0.88)	0.87 (0.85, 0.88)
2008-2011	0.68 (0.66, 0.69)	0.70 (0.68, 0.71)	0.70 (0.69, 0.72)	0.72 (0.70, 0.74)	0.72 (0.71, 0.74)
2012-2014	0.62 (0.61, 0.64)	0.64 (0.62, 0.65)	0.65 (0.63, 0.67)	0.66 (0.64, 0.67)	0.66 (0.64, 0.68)
2015-2018	0.59 (0.57, 0.60)	0.60 (0.58, 0.62)	0.60 (0.58, 0.62)	0.60 (0.59, 0.62)	0.61 (0.59, 0.62)

[a] Reference category are: Income deprivation: 1 (least deprived), Ethnicity: White, Comorbidity: No, Chemotherapy: No, Curative surgery: No, Hormone therapy: No, Radiotherapy: No, Charlson comorbidity index: No, Breast cancer stage: I, Year of diagnosis: 2000-2003. Models were fully adjusted and multiply imputed 10 times as the main analysis.

Table S8. 10-year cumulative risk of overall second primary cancer (SPC) and second primary breast cancer by different definitions

10-year cumulative risk	The least deprived	The most deprived	Difference
**Definition 1 – no exclusions**			
Overall SPC	8.5%	9.5%	1.0%
Breast cancer	2.4%	2.3%	0.1%
**Definition 2 – excluding ipsilateral breast cancers with the same clinical code**			
Overall SPC	8.4%	9.4%	1.0%
Breast cancer	2.4%	2.3%	0.1%
**Definition 3 – excluding ipsilateral breast cancers**			
Overall SPC	7.3%	8.0%	0.7%
Breast cancer	2.1%	2.1%	0
**Definition 4 – excluding breast cancers with the same clinical code (our current definition)**			
Overall SPC	7.6%	8.7%	1.2%
Breast cancer	1.5%	1.5%	0
**Definition 5 – excluding any breast cancers (our current sensitivity analysis 3)**			
Overall SPC (non-breast cancer)	6.1%	7.2%	1.1%

Figure S1. Flowchart of participants selection

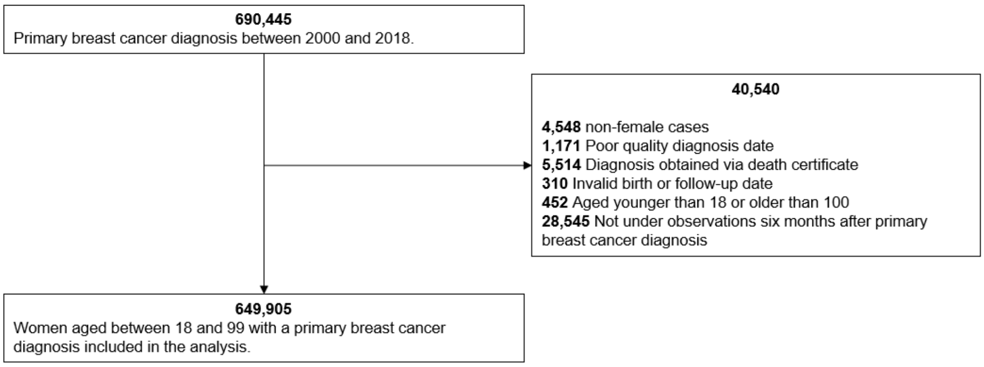

Figure S2. The non-parametric cumulative incidence of second primary cancer of selected sites in the least and most deprived women with breast cancer

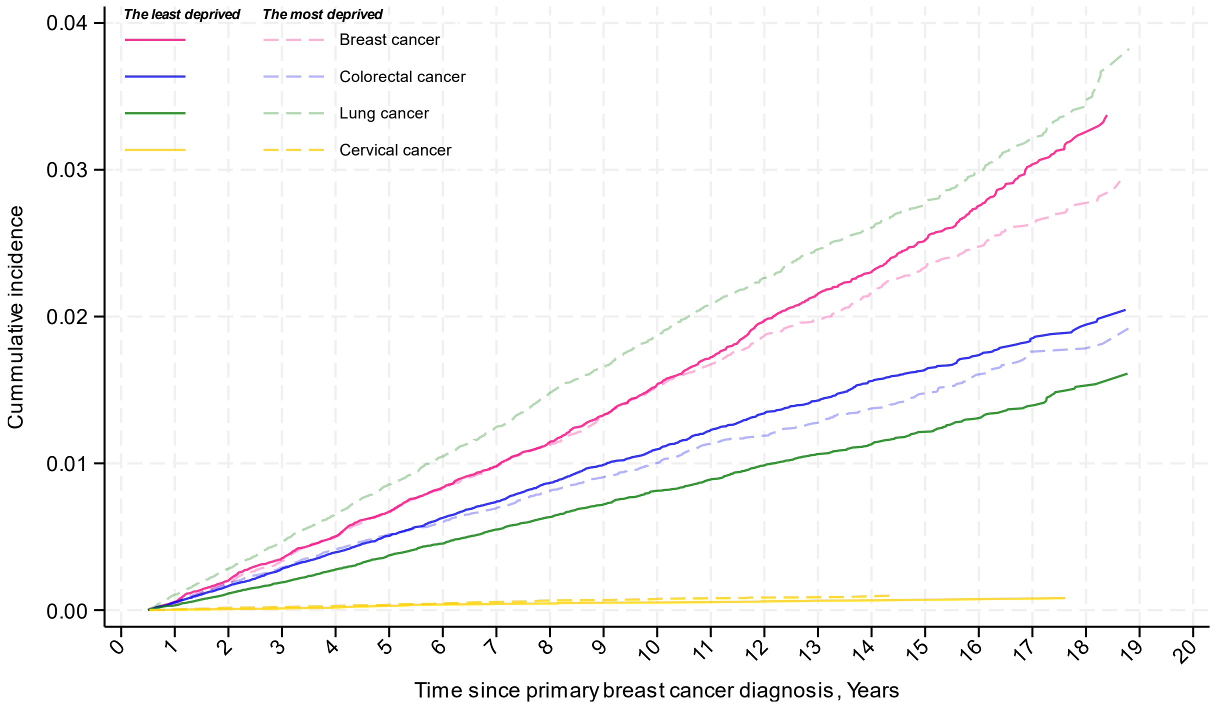

Death and other types of second primary cancer were considered as the competing events.

Figure S3. The standardised cumulative incidence of second primary cancer incidence and death for models including women with complete data on ethnicity and women with complete data on ethnicity and breast cancer stage.

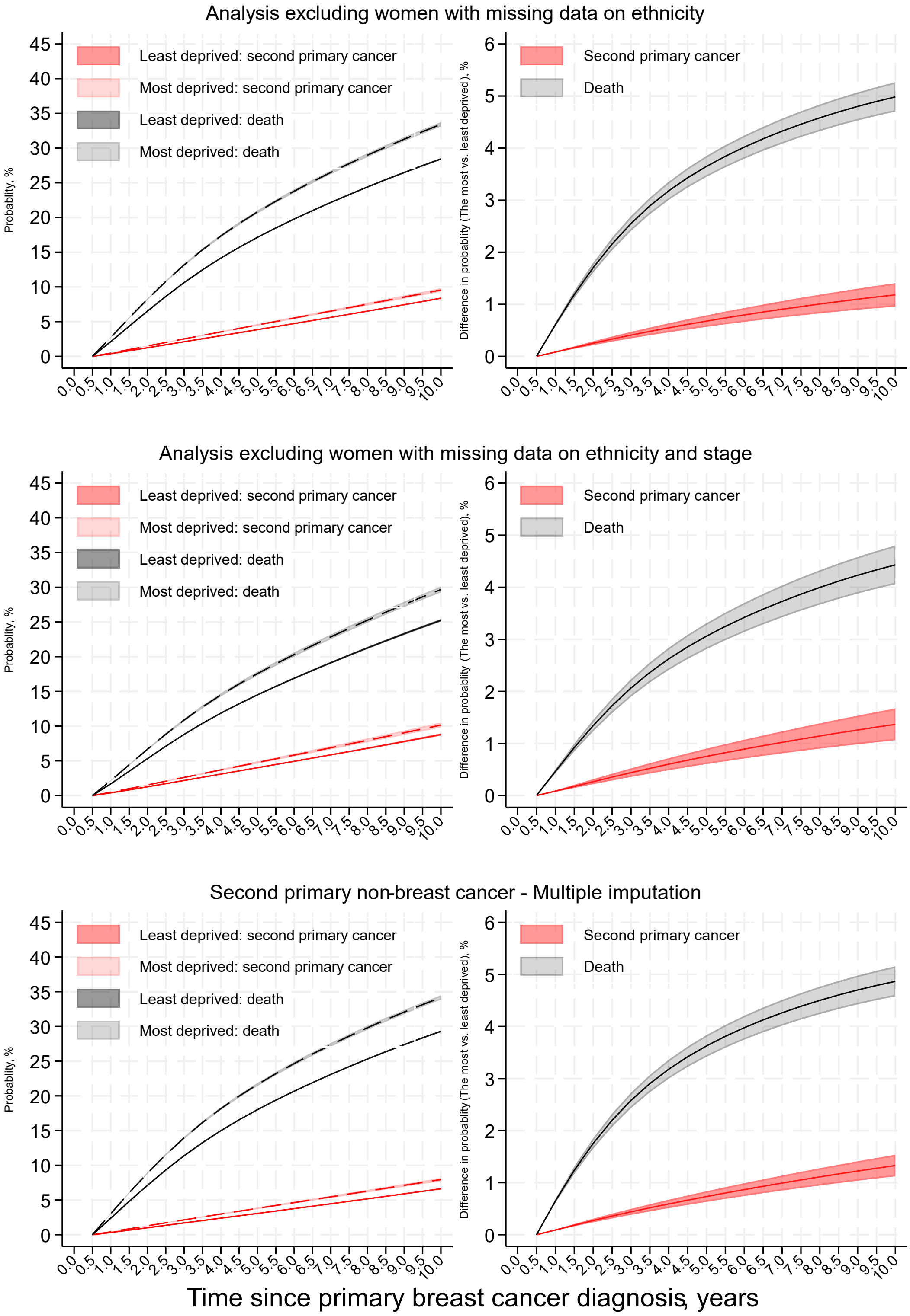

Cumulative incidence of second primary cancer and death were standardised after flexible parametric models adjusting for restricted cubic spline transformed age, ethnicity, year of primary breast cancer diagnosis group, comorbidity, primary breast cancer stage, surgery, chemotherapy, radiotherapy, and hormone therapy. Fully adjusted sensitivity analysis 1: excluding women with missing data on ethnicity and grouping missing stage as a new category. Fully adjusted sensitivity analysis 2: complete case analysis by excluding women with missing data on ethnicity and primary breast cancer stage. Fully adjusted sensitivity analysis 3: the primary outcome only included second primary non-breast cancer (MI analysis), where Multiple imputation (10 times) was performed to account for missing data ethnicity and stage, and all estimates were combined with Rubin’s rules.
